# Pregnancy and Cancer: the INCIP Project

**DOI:** 10.1007/s11912-020-0862-7

**Published:** 2020-02-05

**Authors:** Charlotte Maggen, Vera E. R. A. Wolters, Elyce Cardonick, Monica Fumagalli, Michael J. Halaska, Christianne A. R. Lok, Jorine de Haan, Katrien Van Tornout, Kristel Van Calsteren, Frédéric Amant, for the International Network on Cancer, Infertility and Pregnancy (INCIP)

**Affiliations:** 1 Department of Oncology, KU Leuven, Leuven, Belgium; 2 Department of Obstetrics, University Hospitals Leuven, Leuven, Belgium; 3 Department of Gynecology, Antoni van Leeuwenhoek – Netherlands Cancer Institute, Amsterdam, The Netherlands; 4 Department of Obstetrics and Gynecology, Cooper University Health Care, Camden, NJ USA; 5 Neonatal Intensive Care Unit, Department of Clinical Sciences and Community Health, Fondazione IRCCS Cà Granda Ospedale Maggiore Policlinico, Università degli Studi di Milano, Milan, Italy; 6 Faculty Hospital Kralovske Vinohrady and 3rd Medical, Faculty, Charles University, Prague, Czech Republic; 7 Centre for Gynecological Oncology Amsterdam (CGOA), Antoni van Leeuwenhoek – Netherlands Cancer Institute, Plesmanlaan 121, 1066 CX Amsterdam, The Netherlands; 8 Department of Obstetrics and Gynecology, Amsterdam University Medical Centers, Vrije Universiteit Amsterdam, Amsterdam, The Netherlands; 9 Department of Development and Regeneration, KU Leuven, Leuven, Belgium; 10 Centre for Gynecological Oncology Amsterdam (CGOA), Amsterdam University Medical Centers, Amsterdam, The Netherlands

**Keywords:** Cancer, Pregnancy, Research, Fertility, INCIP

## Abstract

**Purpose of Review:**

Cancer diagnosis in young pregnant women challenges oncological decision-making. The International Network on Cancer, Infertility and Pregnancy (INCIP) aims to build on clinical recommendations based on worldwide collaborative research.

**Recent Findings:**

A pregnancy may complicate diagnostic and therapeutic oncological options, as the unborn child must be protected from potentially hazardous exposures. Pregnant patients should as much as possible be treated as non-pregnant patients, in order to preserve maternal prognosis. Some approaches need adaptations when compared with standard treatment for fetal reasons. Depending on the gestational age, surgery, radiotherapy, and chemotherapy are possible during pregnancy. A multidisciplinary approach is the best guarantee for experience-driven decisions. A setting with a high-risk obstetrical unit is strongly advised to safeguard fetal growth and health. Research wise, the INCIP invests in clinical follow-up of children, as cardiac function, neurodevelopment, cancer occurrence, and fertility theoretically may be affected. Furthermore, parental psychological coping strategies, (epi)genetic alterations, and pathophysiological placental changes secondary to cancer (treatment) are topics of ongoing research.

**Summary:**

Further international research is needed to provide patients diagnosed with cancer during pregnancy with the best individualized management plan to optimize obstetrical and oncological care.

## Introduction

The rarity of cancer in pregnancy complicates patient counselling and decision-making. Historically, most evidence is derived from retrospective, observational data as ethical considerations limit randomized studies. In 2005, encouraged by a young pregnant patient with cervical cancer who was desperate to keep her pregnancy, a medical team from the University Hospitals of Leuven launched a taskforce “Cancer in Pregnancy,” under the umbrella of the European Society of Gynaecological Oncology (ESGO). The lack of knowledge inspired the team to start a unique registry that combines both oncological and obstetric data of women with a cancer diagnosis during pregnancy. Over the years, more differentiated studies in this topic were initiated, and the research group expanded and gained more international interest. As fertility preservation became a point of interest, the research was extended with “fertility in young women with cancer.” In 2014, the taskforce was transformed to the “International Network of Cancer, Infertility and Pregnancy” (INCIP), still supported by ESGO (www.cancerinpregnancy.org). Nowadays this network consists of 67 participating hospitals from 28 countries, which are all compliant with the INCIP study protocol. Meetings are organized to brainstorm on new research topics and discuss ongoing research.

The epidemiology of a cancer diagnosis during pregnancy is difficult to study as nationwide registries do not usually combine both obstetrical and oncological data, resulting in a likely underestimation of the incidence of cancer-related miscarriages or abortions. Also, population-based studies differ in inclusion criteria, often incorporating postnatal cancer diagnoses. Hence, studies focusing solely on cancer during pregnancy report incidence rates of 17 per 100,000 live births and 25–27 per 100,000 pregnancies [1–3]. With the increasing trend to postpone childbirth to a later age, the incidence is expected to increase. The introduction of the non-invasive prenatal (NIP) test to detect major fetal chromosomal abnormalities in obstetrical care results in a further increase of cancer detection (in asymptomatic pregnant patients) [4, 5].

In general, oncological treatment during pregnancy is favored over termination of pregnancy, which has not been shown to improve prognosis, and over elective preterm delivery with its impact on neonatal health [6–8]. Preterm birth, rather than chemotherapy exposure, was found to have an impact on neonatal neurodevelopment [9–11]. Treatment should adhere to protocols presented to non-pregnant women matched for age, offering pregnant women similar prognoses to non-pregnant age-matched women [7, 12, 13]. Chemotherapy can be used during the 2nd and 3rd trimesters until 35 weeks of gestation, with a 3-week therapy-free interval prior to delivery.

**Fig. 1:**
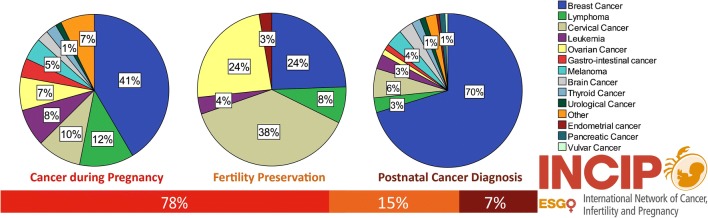
Overview of current registered cases in the INCIP registry (August 2019, *n* = 2653)

The initial aim of the INCIP registry was to provide evidence on obstetric and oncological outcomes of patients with cancer in pregnancy. The most important conclusion of the interim analysis on 1170 patients was that over the past 20 years more patients initiated oncological treatment during pregnancy, resulting in more live births and less preterm births [10••]. Currently, 2059 patients with a cancer diagnosis or oncological treatment during pregnancy, 395 young women with cancer that received fertility preservation, and 199 patients with a cancer diagnosis within 2 years after delivery are registered by the INCIP (Fig. 1). Most patients are registered in Belgium, the Netherlands, Italy, and the USA and one-third of participating centers are non-European. Breast cancer (40%), lymphoma (12%), and cervical cancer (10%) are the most frequent registered cancer types during pregnancy. The majority of patients (67%) received antenatal cancer treatment consisting of surgery (28%), chemotherapy (40%), radiation therapy (1%), targeted therapy (2%), or a combination (28%). Most pregnancies (88%) ended in a live birth, albeit 47% delivered preterm, of which one-third delivered before 34 weeks of gestation. One-fifth of neonates (21%) were small for gestational age (SGA). Congenital malformations were reported in 3% of the live births, which is comparable to the expected rate in the general population.

Current and future collaborations within the INCIP network will provide data that are sufficiently large to formulate recommendations based on solid evidence in order to support physicians and their patients in the challenging clinical decision-making. Here, we discuss current evidence on the most common cancers diagnosed during pregnancy, based on consensus meetings initiated by the INCIP, as well as the future research goals of the INCIP.

## Current Evidence on Management of Cancer in Pregnancy

### Breast Cancer

Pregnancy-induced physiological changes challenge the diagnosis of breast cancer and might delay diagnosis. Hence, women face a 2.5-fold higher risk of being diagnosed at higher stages of disease with inferior oncological prognosis [6]. Approaches to diagnosis differ slightly in that ultrasound is first line during pregnancy rather than proceeding directly to mammography when a patient presents with a palpable mass. Once ultrasound detects a suspicious solid mass, biopsy is performed. Mammography can be performed during pregnancy with a low fetal exposure risk to detect contralateral or multifocal disease, despite limitations of increased parenchymal density found in pregnancy. Gadolinium-enhanced magnetic resonance imaging (MRI) is superior to unenhanced in the evaluation of breast cancers; however, as gadolinium is known to cross the placenta, it is not recommended to perform an enhanced breast MRI during pregnancy [14]. While diffusion-weighted whole-body MRI is a superior alternative to conventional imaging for staging, the diagnostic utility of diffusion-weighted MRI of the breast (without contrast) is still under investigation [15, 16]. Also pineapple juice has been introduced as an alternative contrast agent for diffusion-weighted whole-body MRI without known negative impact on fetal health [17]. Lactating patients can safely continue breastfeeding after gadolinium-enhanced MRI. Review of the literature shows no evidence to suggest that oral ingestion by an infant of the small amount of gadolinium excreted into breast milk would cause toxic effects [18–20].

Sentinel lymph node (SLN) biopsy can be considered during pregnancy instead of standard axillary lymph node dissection for early stage, clinically node-negative breast cancer. Han et al. reported that SLN biopsy during pregnancy had a comparable low axillary recurrence rate as in non-pregnant women and appeared safe for mother and unborn child [11]. The preferred technique is a 1-day protocol using lower doses of 99mTc-labeled albumin nanocolloid without lymphazurin blue dye, which carries a risk for anaphylaxis.

Loibl et al. summarized recent advances in the care of pregnant women with breast cancer [12]. Surgical approach and options remain identical to standards for non-pregnant patients. Radiotherapy is frequently delayed until postpartum, using chemotherapy during the interval between lumpectomy and delivery. Where needed, radiotherapy during early pregnancy can be considered. Chemotherapy should include anthracyclines (doxorubicin or epirubicin) with cyclophosphamide and paclitaxel in sequential sequence, similar to non-pregnant women. Chemotherapy can be started from the 2nd trimester of pregnancy onwards, when organogenesis is completed, until 35 weeks of gestation, with a 3-week therapy-free interval prior to delivery [9, 21]. Trastuzumab, found to affect fetal renal development when used in the 2nd and 3rd trimesters of pregnancy leading to reversible oligohydramnios, is not recommended during pregnancy [22]. Inadvertent exposure in women who conceive while taking trastuzumab does not necessarily require termination of pregnancy since transplacental transfer of IgG molecules only starts after this gestational period [22]. Trastuzumab does not induce congenital malformations though the associated absence of amniotic fluid results in fetal asphyxia and death. Therefore, a single exposure of trastuzumab that does not lead to anhydramnion is not an indication for interruption of pregnancy.

Women with pregnancy-related breast cancer appear to have a similar survival as non-pregnant stage-matched breast cancer patients. Amant et al. showed a similar overall survival (OS) of women with pregnancy-related breast cancer (*n* = 311) and non-pregnant controls (*n* = 865) [23]. In a large Norwegian cohort (*n* = 42,511), no statistically significant difference was observed in cause-specific death between pregnant and non-pregnant breast cancer patients (hazard ratio 1.23, 95% confidence interval (CI) 0.83 to 1.81) [3].

### Gynecological Cancers

The most common gynecological cancers diagnosed during pregnancy are cervical and ovarian cancers, whereas vulvar, vaginal, and endometrial cancers are very rare. Due to the proximity of the tumor to the developing fetus, diagnostic and therapeutic strategies might need to be modified.

Cervical cancer is often diagnosed at an early stage during pregnancy, usually around the 18th week of pregnancy [24•]. The prognosis of the disease seems not to be influenced by the pregnancy itself [24•]. Preoperative evaluation consists of colposcopy, expert ultrasonography, and MRI without gadolinium contrast agent. Computed tomography (CT) and positron emission tomography (PET) should be avoided, although low-dose CT and PET may be considered when results potentially change management or alternative diagnostics are inconclusive.

In early-stage tumors, a pelvic lymphadenectomy is a standard staging procedure, which is feasible during pregnancy until the 22nd week. The choice of laparoscopic or laparotomic approach is based on local expertise; however laparoscopy, if performed in less than 90 min, was found to cause less complications and preterm contractions [25]. Regarding the surgical procedure on the cervix, a simple trachelectomy is preferred to a radical trachelectomy, which has a high rate of obstetrical and surgical complications [26•]. At a more advanced gestational age or in higher stages of disease (IB3, FIGO 2018), neo-adjuvant chemotherapy is the only pregnancy-sparing therapeutic procedure. Neo-adjuvant platinum-based chemotherapy in non-pregnant patients is gaining more attention nowadays [27]. Three-weekly combination of platinum and paclitaxel or a dose-dense combination of platinum with ifosfamide or anthracycline could be used [26•]. When opting for non-pregnancy-sparing management, a radical hysterectomy with the fetus in utero (during the 1st or early 2nd trimester) or after hysterotomy (during the late 2nd trimester) can be performed. Chemoradiation with the fetus in utero can be applied when diagnosed during the 1st trimester (resulting in fetal loss within a few days) while hysterotomy and evacuation of the uterus as a first step is advised when diagnosed during the 2nd trimester [26•]. When cervical cancer is still in situ, a cesarean section is mandatory in order to avoid implants in the episiotomy site. A corporeal incision is advised in order to avoid abdominal wound implants.

Adnexal masses are found in approximately 2–4% of the pregnancies [28]. Ultrasonography is the main diagnostic tool, while tumor markers are less valuable due to gestational changes. Surgical staging, including infracolic omentectomy, appendectomy, pelvic-peritoneal biopsies, lymph node dissection, and frozen section examination if indicated, is ideally performed between the 14th and 22nd weeks. Restaging postpartum should be organized if the Douglas pouch could not be reliably examined. In the case of advanced epithelial tumors, chemotherapy is administered in a neo-adjuvant setting since cytoreduction to no residual tumor is not feasible during pregnancy. During pregnancy, a higher frequency of non-epithelial tumors is being found due to the younger age of patients. These cases could often be treated by pregnancy-preserving procedures.

Only a few cases of vulvar cancer have been reported, usually diagnosed at an early stage. Standard treatment consists of radical local excision with a dissection of unilateral or bilateral lymph node dissection. If indicated, a SLN biopsy using technetium in a short-term protocol or indocyanine green can be performed in pregnancy [26•].

### Hematological Cancers

Lymphomas are more commonly diagnosed during pregnancy than leukemia, with an estimated incidence of one in 6000 and one in 75,000–100,000 pregnancies respectively [29]. As in general, Hodgkin and non-Hodgkin lymphomas should be treated as in non-pregnant patients [30•]. Depending on maternal symptoms, cancer stage, and aggressiveness of the hematological entity, immediate cytotoxic treatment, or a deferral of treatment until after delivery in well-selected cases, can be considered. Chemotherapeutic agents, especially when administered in combination, should be avoided during the 1st trimester. Commonly used regimens for lymphomas (doxorubicin, bleomycin, vincristine, dacarbazine (ABVD) or cyclophosphamide, hydroxydaunorubicin, oncovin, prednisone (CHOP)) can be considered from the 2nd trimester of pregnancy with regular obstetrical follow-up to safeguard fetal growth [29, 31•]. Aviles et al. reported on reassuring outcome of 54 newborn babies prenatally exposed to chemotherapy for hematological malignancies during the 1st trimester of pregnancy [32]. Data of this series can be useful in counselling patients who were accidentally exposed to cytotoxic drugs in pregnancy. However, early pregnancy is a very vulnerable period as fetal organogenesis takes place and chemotherapy during the 1st trimester of pregnancy should be avoided if maternal condition allows, until more safety data are available. There is some experience with rituximab, an anti-CD20 monoclonal antibody, that is commonly used for the treatment of non-Hodgkin lymphoma and its use might be considered from the 2nd trimester onwards [33]. In a large retrospective observational study, progression-free and overall survival for 77 patients with HL during pregnancy was not significantly different from 211 non-pregnant patients matched for stage and prognostic scores [31•].

Leukemia in pregnancy presents a very difficult challenge and initiation of treatment should not be delayed in order to avoid any impact on maternal prognosis. In early pregnancy, termination is often unavoidable to conserve maternal prognosis, given the teratogenicity of antineoplastic agents, and the potential for maternal complications during periods of extreme pancytopenia and immunosuppression. In these cases, induction chemotherapy prior to termination of pregnancy sometimes needs to be considered in order to allow a safe termination. Leukapheresis can be considered during pregnancy and is indicated in the presence of significant leukocytosis- and leukostasis-related complications, such as vascular occlusion. Thromboprophylaxis should be considered especially in pregnant patients with leukemia as the hypercoagulable state of pregnancy can be aggravated by myeloproliferative neoplasms.

## Current Evidence on Pharmacokinetics

Physiological gestational changes can influence all pharmacokinetic parameters. A significant increase in plasma volume, extracellular water, and body fat can change the distribution volume [34]. Additionally, a decrease in plasma protein concentrations, an increased glomerular filtration and changes in hepatic metabolizing enzyme activity have been reported in pregnancy. This gestational effect is expected to be most pronounced in the 3rd trimester of pregnancy. These altered pharmacokinetics potentially have an effect on both the efficacy and safety of drug treatment.

Data on pharmacokinetics of chemotherapy during pregnancy are scarce and current evidence is based on relatively small case series. Compared with non-pregnant patients, a decreased area under the curve (AUC), decreased peak plasma concentration, and an increased distribution volume have been reported (epirubicin, doxorubicin, docetaxel, and paclitaxel) [35, 36]. This effect appears to be more apparent for taxanes than for anthracyclines. Yet, there is no evidence of inferior survival of patients with a cancer diagnosis or treatment during pregnancy [3, 23, 31•]. Thus, current recommendations advice to prescribe chemotherapy based on the actual body weight of pregnant women and do not support gestation-related dose adaptations of cytotoxic drugs [12, 26•, 35].

## Current Evidence on Outcome of Children and Ongoing Research

An increased awareness of the feasibility of cancer treatment during pregnancy resulted in more pregnant women receiving antenatal treatment and more live births [10••]. Indeed, chemotherapy administered after the 1st trimester of pregnancy does not cause fetal malformations, but it might increase the risk of neonatal complications [8, 10••]. Prematurity is the most common adverse outcome reported. It represents the main determinant for early postnatal complications as well as impaired neurodevelopmental outcome, with the most immature infants bearing the highest risk [8, 37–42]. Early neonatal complications include neonatal death, neonatal intensive care unit (NICU) admission, SGA, hematologic disturbances, and prematurity-related disorders (respiratory distress syndrome, metabolic disturbances, sepsis, jaundice, and necrotizing enterocolitis) [10••, 11••, 12, 13, 38, 43–45]. In the cancer in pregnancy population, preterm birth is likely to be medically induced for maternal oncological reasons. However, during the last two decades, a reduction of iatrogenic preterm deliveries (relative risk (RR) 0.91, IC 0.84–0.98) has been reported [10••].

Although evidence is still controversial, SGA seems to be more common after cancer in pregnancy, especially when chemotherapy is administered during pregnancy. An increasing incidence of SGA in chemotherapy-exposed newborns has been reported, which might expose the infants to an increased risk of perinatal mortality and morbidity [8, 10••, 32, 37, 40, 42, 43]. Interestingly, few data are available on the real incidence of intrauterine growth restriction (IUGR). Yet, IUGR infants carry the highest risk for short- and long-term complications. Initiating chemotherapy before 15 weeks of gestation appears to significantly increase the risk for IUGR (OR 3.0) [46]. But treatment delay for fetal protection should always be balanced against the maternal risk of postponing chemotherapy. Other factors, such as maternal stress, the nutritional state, and the disease itself, may contribute to the observed effect. This observation highlights the need of careful biometric and umbilical and brain Doppler surveillance, especially during and after chemotherapy administration.

Transient hematotoxicity is a potentially serious, although rare, neonatal side effect of chemotherapy administered to the mother [43]; therefore, a 3-week window between the last cycle of chemotherapy and delivery is suggested to allow fetal bone marrow recovery. A recent study investigated the incidence of neonatal leukopenia (white blood cells < 5000/mm^3^; 3%) and neutropenia (neutrophils < 1000 mm^3^; 7%) after chemotherapy exposure; neutropenia was more common when chemotherapy was given 22 to 28 days before birth, while the risk of leukopenia was highest if delivery occurred less than 7 days from chemotherapy [21].

Among long-term complications, neurodevelopmental impairment, cardiotoxicity, ototoxicity, endocrine disorders, and secondary malignancy have been reported. Concerns still exist on the potential detrimental effects of maternal cancer treatment on the fetal brain and subsequent neurocognitive impairments. The developing brain remains vulnerable throughout the whole pregnancy, especially during the last trimester in which multiple crucial biological processes occur. No congenital brain malformations or acquired brain lesions were observed and no significant differences were found in both regional and total brain volumes of infants prenatally exposed to chemotherapy (*n* = 21; 81% epirubicin) compared with control infants matched for gestation at birth [47].

Reassuring neurocognitive outcomes have been reported in children born to mothers with cancer, mainly by retrospective cohort studies [32, 42, 48, 49]. In 2015, Amant et al. reported no effect of cancer treatment on neurobehavioral performances at 36 months, although an independent effect of prematurity on cognitive outcome was demonstrated [8]. Longer follow-up data on cognitive, behavioral, and academic performances are necessary considering the known “chemo-brain” effect. Impairment in attention and executive functions has been described in childhood cancer survivors treated with chemotherapy [50].

According to specific drug exposure, surveillance on long-term cardiotoxicity (after anthracyclines exposure) and hearing impairment (cisplatin) is recommended. However, the evidence regarding cardiac function on children prenatally exposed to anthracyclines is reassuring: non-clinically relevant differences in echocardiographic findings have been reported in exposed children compared with non-exposed ones [8, 42, 51, 52]. Concerns related to hearing function throughout infancy are mainly based on data from young cancer survivors [53, 54]. However, on indication, the use of carboplatinum during pregnancy is preferred, as there are case reports of impaired hearing in children prenatally exposed to cisplatin [8, 41, 55]. The occurrence of endocrine disturbances as well as secondary malignancy needs to be investigated in long-term cohort studies.

## Current Evidence on Psychological Coping Strategies and Ongoing Research

When future parents are confronted with a cancer diagnosis during pregnancy, they will experience a rollercoaster of ambiguous emotions; the joy of being pregnant and motherhood might become overwhelmed by fear for the lives of the mother and unborn child. In a retrospective study, 21–52% (based on two self-administered psychological questionnaires) of 74 pregnant women with a cancer diagnosis self-reported significant levels of distress, which is higher than published rates in healthy pregnant women (15%) and breast cancer patients (20–40%) [56–58]. The INCIP retrospectively investigated different coping strategies for patients and their partners by questionnaires (Cognitive Emotion Regulation Questionnaire (CERQ)) and a newly constructed Cancer and Pregnancy questionnaire [59]. One might use three different coping strategies: positive coping, internalizing coping, and blaming. Couples using internalizing coping strategies deal with the highest levels of distress and may benefit from additional psychosocial support. Ongoing research aims to validate the Cancer and Pregnancy questionnaire, in order to implement it in clinical practice as a tool for healthcare workers to provide patient-oriented psychological support.

## Current Evidence on Placental Aspects and Ongoing Research

The risk of SGA in cancer in pregnancy is two- to threefold higher than that in the general population, especially after administration of platinum-based agents [10••]. Deficient placental nutrient and oxygen supply account for up to 90% of all SGA pregnancies in the general population [60, 61]. Maternal cytotoxic treatment penetrates the placenta and subsequently might affect placental development and fetal growth. Only a limited number of (mainly animal) studies have been focusing on placental changes due to maternal cancer (treatment). The placental weight of untreated tumor-bearing animals is significantly reduced in comparison with non-tumor-bearing controls and morphologic differences have been observed [62, 63]. Also an increased level of oxidative stress and an affected DNA repair system in placental tissue have been noticed [62, 64]. To date, only two studies have been evaluating chemotherapy-related placental changes in women. The first showing non-specific placental findings including villous hypoplasia (*n* = 13) and the second confirming enhanced oxidative damage (*n* = 25) [65, 66]. Genetic changes have been only studied after exposure to alkylating agents in non-tumor-bearing animal models, confirming dose-dependent DNA adduct levels in placental tissue and methylation of placental DNA, endorsing the oncogenic character of these drugs [67, 68]. Placental irradiation effects have only been studied in one mice study, showing no morphological changes, but reduced placental weights and significantly altered expression of genes related to oxidative damage [69].

Placental metastases are mainly reported in women with malignant melanoma (22–50%) and are assumed to be hematogenously disseminated [70–74]. They typically occur in women with otherwise metastatic disease [72]. Due to a suggested placental barrier between the maternal and fetal interface, placental metastases are mainly located in the intervillous space and to a lesser extent in the villi [75]. Fetal involvement is rare and always preceded by villous invasion of maternal cancer cells [75]. Histopathological placental examination is crucial in identifying potential fetal involvement and should be strongly considered in women with a cancer diagnosis, especially in a metastasized setting. Limited knowledge about placental changes has led to the implementation of the placenta study in order to study cancer (treatment) effects. Placental (epi)genetic changes and subsequent long-term risks to these infants are subject of ongoing studies. Also, studies on meconium of infants, who were prenatally exposed to an expanded panel of chemotherapeutic agents and metabolites, after a successful detection of paclitaxel and metabolites, further explore direct fetal exposure [76].

## Importance of a Multidisciplinary Advisory Board and Development of a Clinical Pathway for Cancer and Pregnancy

**Fig. 2:**
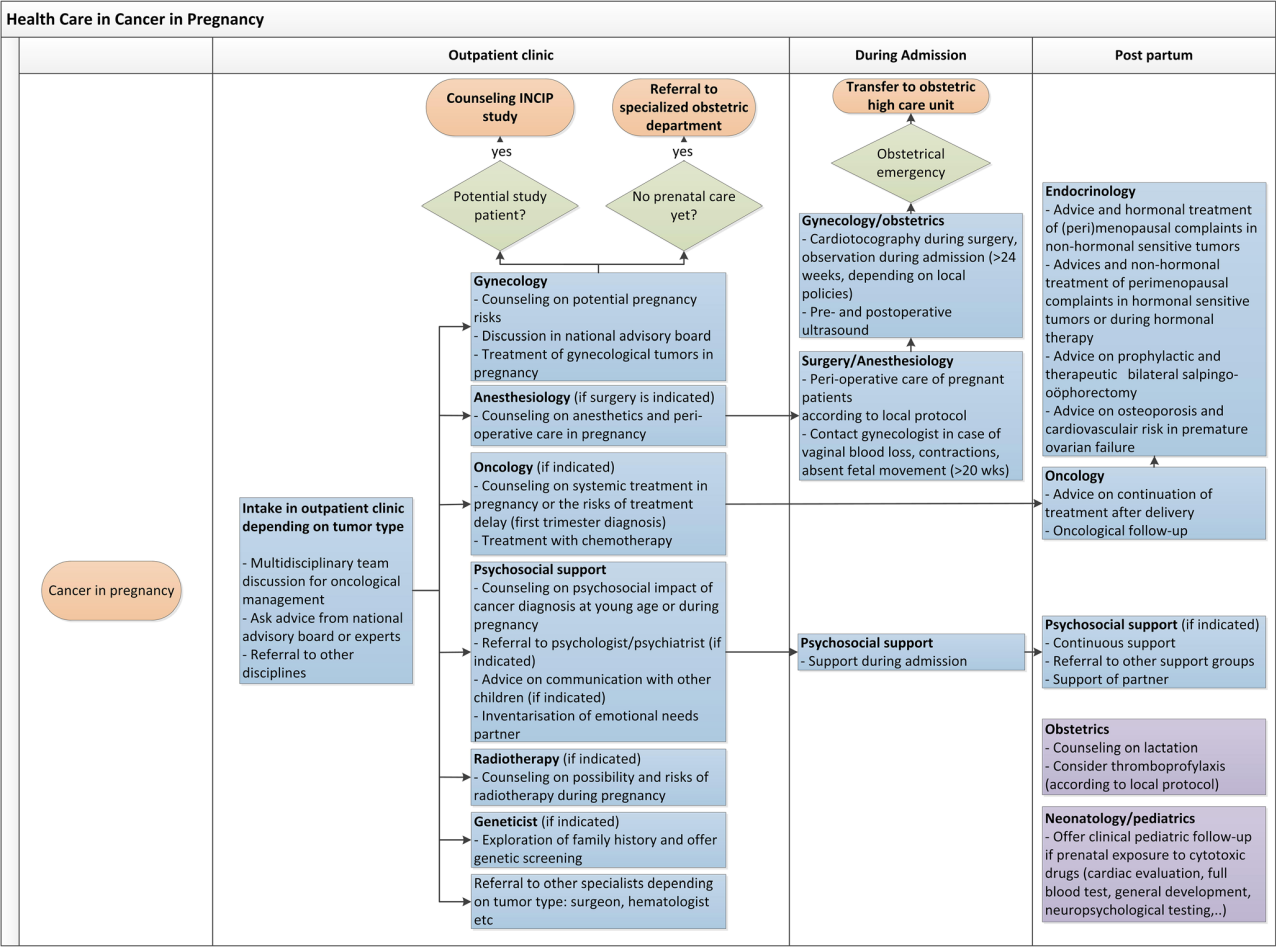
Multidisciplinary clinical care pathway for patients confronted with a cancer diagnosis during pregnancy

Even though the co-incidence of cancer and pregnancy is slowly increasing, it is still a rare event. The consequence is that individual doctors see less than one pregnant patient with cancer a year. Adding the heterogenic character of the population with all the different types of cancer occurring at different gestational ages, an experienced multidisciplinary team is indispensable in order to provide optimal care for both mother and child. This team should at least include a medical oncologist, perinatologist, gynecologic oncologist, pediatrician, radiotherapist, and psychologist or social worker. Other valuable members are a geneticist and clinical pharmacologist. Depending on the type of cancer, a surgeon, hematologist, or other specialists should be added (Fig. 2).

As defined by the European Reference Networks (ERNs) for rare diseases, a disease is considered rare if the number of affected people is less than 1 in 2000 [77]. This implies that cancer in pregnancy qualifies as a rare disease. The care for patients with a rare disease is complicated by the small number of patients, lack of evidence-based diagnostic and treatment options, logistic problems due to the scattering of patients across countries, and limited clinical expertise. It is widely accepted that increased exposure of a medical specialist to a rare health problem improves knowledge and quality of care [78].

With the expanding number of experienced healthcare professionals, the INCIP could theoretically act as an ERN that connects healthcare professionals in Europe. Representatives of the ERN would collect their expertise into national advisory boards.

In the Netherlands, a first step in realizing a reference network has been made in 2012, when the national “Advisory Board on Cancer in Pregnancy” was established. This advisory board consists of healthcare professionals from different hospitals and covers most of the specialisms involved in the care of pregnant cancer patients. All involved professionals have their own expertise and are available for consultation by other healthcare professionals confronted with a pregnant patient with cancer. The aim is to advise healthcare professionals on optimal care, based on up-to-date information about the possibilities and risks of diagnostics and treatment. The board will advise referral to another hospital if specific expertise is required and aims to include these patients into the ongoing INCIP research projects.

International advisory boards on specific malignancies would require digital support with easy access to all available recommendations and links to research projects, specialized national referral hospitals, and patient information. Consensus meetings can aid the establishment of up-to-date and experience-based guidelines. Finally, (inter)national boards can facilitate an e-learning or interactive online course to increase knowledge and awareness on cancer in pregnancy, leading to improved care and possible improved outcome.

## Conclusion

With the establishment of INCIP, a crucial step has been made in the improvement of care for pregnant cancer patients and their offspring. Most of the current knowledge is based on voluntary participation of patients treated by INCIP members. With a rapidly evolving field like oncology in combination with a rare and heterogenic population, the need for further expansion of the INCIP research projects is of the utmost importance to provide these patients with the best possible knowledge and reduce adverse outcomes. We therefore invite all healthcare professionals confronted with pregnant cancer patients to contact national experts or the INCIP to gain up-to-date knowledge on their specific situation and, in addition, to establish a national advisory board and participate in INCIP research projects (www.cancerinpregnancy.org).
